# Synthesising participants’ accounts from different mental health trials: the stride study

**DOI:** 10.1186/1745-6215-16-S2-O40

**Published:** 2015-11-16

**Authors:** Katrina Turner, John Percival, David Kessler, Jenny Donovan

**Affiliations:** 1 University of Bristol, Bristol, UK

## 

Researchers are increasingly utilising opportunities to bring together data from different qualitative studies in order to compare across data sets. However, limited guidance exists on how to do this. This presentation details the approach we used when synthesising four data sets in a study comparing patients’ experiences of different treatment modalities for depression.

All four studies entailed conducting in-depth interviews with participants diagnosed with depression and were nested within large, primary care depression trials. Interventions evaluated within the trials included antidepressants, listening visits, CBT and facilitated physical activity.

Data were analysed to explore what patients valued in their relationships with practitioners. This focus arose inductively from reading and re-reading transcripts. 35 transcripts were analysed using a coding frame that reflected emerging themes and which could be applied across the transcripts. The analysis established the extent to which themes were evident within the combined data set, and examined differences and commonalities between the four data sets.

We found that patients receiving different treatments valued the same key practitioner attributes. Thus, through synthesising multiple data sets, we gained new insights and identified commonalities which increased confidence in the conclusions drawn. The limitations of our work related to those which had existed in the original studies, e.g. few participants from ethnic minorities. The success of the study related the selection of the four data sets (all were interview-based), the inductive approach used to identify the focus of our analysis, and the use of generic codes which enabled comparisons to be made.

